# Pestivirus infection in reindeer (*Rangifer tarandus*)

**DOI:** 10.3389/fmicb.2015.01187

**Published:** 2015-10-26

**Authors:** Magdalena Larska

**Affiliations:** Department of Virology, National Veterinary Research InstitutePuławy, Poland

**Keywords:** pestivirus, BVDV, BDV, reindeer, caribou

## Abstract

Reindeer species (*Rangifer tarandus*, Linnaeus, 1758) includes wild and semi-domesticated ruminants belonging to *Capreaolinae* subfamily of *Cervidae* family reared in Eurasia (reindeer subspecies) and North America (caribou subspecies). Herding of reindeer has a great historical, socio-economic and ecological importance, especially to indigenous ethnic minorities. Infectious disease threats may therefore impact not solely the animal population driving it to further extinction and irreversible alterations to the wild environments of northern hemisphere, but also add to cultural changes observed as negative impact of globalization. Introduction of new technologies to control of reindeer migration between dwindling pasture areas and intensification of reindeer husbandry may facilitate the intra- and interspecies transmission of pathogens. The role of the reindeer as a potential BVDV reservoir has been studied, however, the number of publications is rather limited. The observed seroprevalences of the virus varied significantly between different geographical regions with different epidemiological situation. Most frequently limited number of animals studied and the differences in the sensitivities and specificities of the diagnostic test used could have also influenced on the differences between the studies. No pestivirus has been ever detected in free-ranging reindeer, however, a putative pestivirus strain named V60-Krefeld has been isolated from reindeer kept at a German Zoo in the 1990’s. The virus was characterized as border disease virus type 2 (BDV-2) closely related to German ovine strains. The cross-neutralization studies of the semi-domesticated reindeer sera from Sweden suggested infection with a strain related to BDV-1 or BDV-2. The available data indicates that reindeer might be infected by a endemic species-specific BDV-like strain. However, the interspecies transmission of BVDV from domestic animals should not be excluded, since the susceptibility of reindeer to BVDV-1 has been confirmed under experimental conditions.

## Reindeer Husbandry

Reindeer are cervids, which belong to *Rangifer tarandus* species (Linnaeus, 1758), subfamily *Capreaolinae* (New Word, telemetecarpal deer, Brookes, 1928). The worldwide population is estimated at more than four million animals of several main subspecies occupying different regions including Eurasian reindeer (*R. t. tarandus*) and caribou inhabiting Canada, U.S. (*R. t. caribou*) and Alaska (*R. t. granti*). Reindeer subspecies differ greatly in their size with the largest being the woodland caribou and the smallest being the wild Svalbard reindeer (*R. t. platyrhynchus*, Vrolik, 1829) from Norwegian archipelago of Svalbard (with the largest island of Spitzbergen). The circumpolar distribution of reindeer determines their diet, migration potential and reproduction specificity. As the only mammals, they are able to survive winter by feeding only on lichen (*Cladonia rangiferina*). They breed seasonally, however, their reproduction cycle may adapt to climate changes and access to food. The greatest threat to reindeer are predators responsible for the high mortality in particular among the youngest animals (Tryland, 2012). The alterations of the climate, which may influence winter lichen pastures lead to the necessity of supplementary feeding. Additionally, fragmentation of pasture areas disrupts natural migration routes and increases the need of transport of reindeer by lorries and usage of motor vehicles such as four-wheel drive cars, snow scooters and helicopters in their herding (Tryland, 2012). Modernization, changes of reindeer management, gathering of animals for censusing or sorting for slaughter, ear tagging, and veterinary care increases animal density and stress, and finally can lead to increased risk of transmission of pathogens (Malmfors and Wiklund, 1996).

Reindeer were reported to suffer from foot-and-mouth disease (FMD), necrobacillosis, pasteurellosis, anthrax, brucellosis, paratuberculosis, infectious keratoconjunctivitis (IKC) and contagious ecthyma (Gavier-Widen et al., 2012; Tryland, 2012). Some of the pathogens are endemic in reindeer such as Cervid herpesvirus 2 (CvHV2; das Neves et al., 2010), CvHV3 and *R. tarandus* papillomaviruses 1 and 2 (RtPV1, RtPV2) associated with IKC (Smits et al., 2013). Despite the limited number of studies on pestivirus infections in reindeer, they provide a compelling evidence of wide spread of these viruses in many populations.

## Pestivirus Epidemiology in Reindeer

Epidemiological data of pestivirus infections in the species of reindeer are limited to only a few studies, which were performed mostly at the end of last century (**Table 1**). Based on specific antibody presence, pestivirus infections were confirmed in the reindeer on two continents, Europe and North America. Since in all the serosurveys bovine viral diarrhea virus type 1 (BVDV-1) strains were used as a reference, the results indicated *de facto* BVDV seroprevalences. Only Kautto et al. (2012) have tried to establish the pestivirus strain responsible for the infections in reindeer in Sweden. The percentages of BVDV seropositive reindeer varied from 0% in the wild Svalbard reindeer of Norway (Stuen et al., 1993) and in the four populations of Swedish reindeer tested in the 1980’s (Rehbinder et al., 1985) to nearly 70% in the migratory George River caribou from Canada (Elazhary et al., 1981). The seroprevalence has been associated with variables such as herd, age, and bovine herpesvirus type 1 (BoHV1) seropositivity (Rehbinder et al., 1992; Stuen et al., 1993; Lillehaug et al., 2003; Kautto et al., 2012). The reindeer or domestic ruminant densities have not been shown to explain the variation of herd seroprevalences. The differences in the herding techniques (extensive or intensive), management or the possible presence of persistently infected (PI) animals were more likely risk factors (Kautto et al., 2012). Similarly to many other infectious diseases, the risk of infection increased with the age, resulting in a significantly higher pestivirus seroprevalence in adult reindeer in comparison to calves (Stuen et al., 1993; Kautto et al., 2012). The possible interactions between pestiviruses and alphaherpesviruses has been observed in reindeer (Stuen et al., 1993; Tryland et al., 2005; Kautto et al., 2012), which by analogy to cattle (Kampa et al., 2009), could reflect the immunosuppressive nature of both virus groups.

**Table 1:** A summary of the literature on pestivirus seroprevalence in the reindeer species (*Rangifer tarandus*).

Origin	Year	Reindeer subspecies	n/N^∗^	Pestivirus seroprevalence	Serological test used^∗∗^	Association	Reference
Svalbard, Norway Finnmark, Norway	1990 1991	*R. t. platyrhynchus R. t. tarandus*	0/40 54/326	0% 17%	VNT against NADL strain BVDV	• Age (calves: 6%; adults: 41%) • Herd (0–40%) • Concurrent seropositivity to BoHV1 (27% in adults; 1% in calves)
Stuen et al., 1993
Southern Norway	1993–2000	*R. t. tarandus*	34/810	4.2%	VNT against NADL and Norwegian MD2154/66 strains	• Population or area (0–51%)
Lillehaug et al., 2003
Finnmark, Norway	2000	*R. t. tarandus*	14/43	33%	VNT against NADL strain BVDV	• Concurrent seropositivity to BoHV1 (50%)
Tryland et al., 2005
Northern Sweden	1973–1982	*R. t. tarandus*	3/50	6%	In-house ELISA	• Herd (0-25%)
Rehbinder et al., 1992
Northern Sweden	1980	*R. t. tarandus*	0/26	0%	VNT against Ug-59 strain BVDV	No data	Rehbinder et al., 1985
Northern Sweden	2001–2002	*R. t. tarandus*	408/1158	35.2%	Commercial BVDV ELISA and VNT against six pestiviruses	• Age (calves: 19.2%; adults: 48.9%) • Concurrent seropositivity to BoHV1 (31%)
Kautto et al., 2012
Quebec, Canada	1978 1979	*R. t. caribou*	21/30 17/28	70% 60.7%	VNT against Oregon C24V strain BVDV	No data	Elazhary et al., 1981
Alaska, U.S.	1978–1981	*R. t. granti*	2/67	3%	VNT against Oregon C24V strain BVDV	No data	Zarnke, 1983

*^∗^number seropositive/number animals tested; ^∗∗^VNT, virus neutralization test*.

The questions on the pestivirus strain and the source of infection in reindeer have been raised by some authors. Except for Kautto et al. (2012), only serological test based on BVDV-1 strains isolated from cattle were used. However, it is very likely that, as in case of alphaherpesviruses, the pestivirus circulating in reindeer is specific to the species (das Neves et al., 2010). The Swedish group (Kautto et al., 2012) have found that BVDV ELISA positive reindeer sera reacted with the highest titers to border disease virus 1 (BDV-1) 137/4 strain and with relatively lower titers to BDV-2 V-60 strain isolated from reindeer at German Zoo (Becher et al., 1999, 2003; Avalos-Ramirez et al., 2001). Some cross-reactivity toward BVDV-1 NADL strain was also observed, however, it was connected to the wider cross-reactivity of BVDV-1 (Becher et al., 2003). The study failed to demonstrate, which type was actually circulating in the reindeer population studied, however, it suggested BDV strain rather than a bovine pestivirus. Another convincing argument for independent infection process among reindeer and caribou was the lack of direct contact with domestic ruminants discussed by Elazhary et al. (1981) and Kautto et al. (2012), respectively. Rehbinder et al. (1992) concluded that closer contact of reindeer with domestic ruminants has not affected BVDV seroprevalence. The transmission of BVDV between cattle and reindeer by blood feeding flies has been suggested (Rehbinder et al., 1992). The virus was detected in the insects fed on PI calves (Tarry et al., 1991), however, the exposed BVDV naïve calves remained virus-free and did not seroconvert throughout the study (Chamorro et al., 2011). The transmission from cattle to reindeer was also found unlikely in Norway. The highest pestivirus seroprevalence was found in the extreme northern county of Finnmark where the density of domestic ruminants is very low (Stuen et al., 1993; Lillehaug et al., 2003). Additionally, most cattle in northern Scandinavia where reindeer are reared remains BVDV free after successful eradication plans implemented already in 1990’s in Norway (Løken and Nyberg, 2013), Sweden (Hult and Lindberg, 2005), and Finland (Rikula et al., 2005).

## Pestivirus Isolation and Pathogenicity in Reindeer

The susceptibility of reindeer to BVDV-1 infection has been experimentally shown (Morton et al., 1990). The clinical signs included mucous and bloody diarrhea, transient laminitis, and coronitis, leucopenia, serous to mucopurulent nasal discharge. However, no pestivirus has been isolated from wild or semi-domesticated reindeer (Rehbinder et al., 1985; Rockborn et al., 1990; Kautto et al., 2012). The cases of conjunctivitis, ulcerative and necrotizing lesions of nose and oral mucosa in Swedish reindeer could not have been linked to BVDV infection (Rehbinder et al., 1985; Rockborn et al., 1990). The presence of pestivirus genome was also investigated in nearly 280 healthy slaughtered seronegative reindeer from the districts with up to 98% of virus seroprevalence using pan-pestivirus real-time RT-PCR (Kautto et al., 2012). No viral RNA was found in the sera of those animals.

So far the only pestivirus isolated from *R. tarandus* species was V60-Krefeld (Reindeer-1) strain obtained from reindeer, which died with signs of severe diarrhea and anorexia at Duisburg Zoo in Germany in 1996 (Becher et al., 1999; Giangaspero et al., 2006). The outbreak affected all reindeer in the zoo herd as well as an European bison (*Bison bonasus*) from which another, closely related strain V65-Krefeld (Bison-1) was isolated (Becher et al., 1999; Giangaspero et al., 2006). Initially, the reindeer strain has been included as a separate novel species within the genus *Pestivirus* (Avalos-Ramirez et al., 2001). Further phylogenetic studies based on N^Pro^ and E2 coding sequences revealed that V60 and V65 strains were most closely related to border disease virus type 2 (BDV-2) strains isolated from German sheep in 1999 and 2000 (Becher et al., 1999, 2003). The V60 strain grew efficiently in Madin-Darby bovine kidney (MDBK) cells with titers reaching 10^6^–10^7^ TCID_50_ after four consecutive passages (Giangaspero et al., 2006). The antiserum against V60 strain preferably neutralized BDV-like and classical swine fever virus (CSFV) strains, but did not react with BVDV-1 strains NY-1 and C86 (Avalos-Ramirez et al., 2001; Becher et al., 2003). If the pestivirus isolated from zoo reindeer is related to the virus circulating among free-living animals, which is very likely judging by the results of Kautto et al. (2012), the seroprevalences estimated using tests based on BVDV-1 strains may be underestimated.

In conclusion, the few reports on the distribution of pestivirus in reindeer has shown great variation of seroprevalences depending on the time of the study, geographical origin, animal age, health status, and epidemiological situation of other endemic infections. Pestivirus infection in free-ranging reindeer is more likely to be independent from domestic ruminants, mainly due to the circumpolar distribution of reindeer and therefore limited contact between the species, or pestivirus eradication in far animals as in Scandinavia. Some studies suggest that the pestivirus strain circulating in reindeer is probably a species-specific BDV-like virus.

## Conflict of Interest Statement

The author declares that the research was conducted in the absence of any commercial or financial relationships that could be construed as a potential conflict of interest.
